# Vagal Blocking Improves Glycemic Control and Elevated Blood Pressure in Obese Subjects with Type 2 Diabetes Mellitus

**DOI:** 10.1155/2013/245683

**Published:** 2013-07-30

**Authors:** S. Shikora, J. Toouli, M. F. Herrera, B. Kulseng, H. Zulewski, R. Brancatisano, L. Kow, J. P. Pantoja, G. Johnsen, A. Brancatisano, K. S. Tweden, M. B. Knudson, C. J. Billington

**Affiliations:** ^1^Brigham and Women's Hospital, 75 Francis Street, Boston, MA 02115, USA; ^2^Adelaide Bariatric Center, Flinders Private Hospital, Suite 502/Level 5, Bedford Park, SA, 5041, Australia; ^3^Instituto Nacional de la Nutricion, Salvador Zubiran (INNSZ), Vasco de Quiroga 15, Tlalpan, 1400 Mexico City, DF, Mexico; ^4^Center for Obesity, St. Olavs Hospital, Olav Kyrres Gate 6, 7006 Trondheim, Norway; ^5^Division of Gastroenterology, University Hospital Basel, 4031 Basel, Switzerland; ^6^Institute of Weight Control, 495 Windsor Road, Baulkham Hills, NSW 2153, Australia; ^7^EnteroMedics Inc., 2800 Patton Road, St. Paul, MN 55113, USA; ^8^University of Minnesota, Minneapolis, Minnesota Veterans' Administration Medical Center, One Veterans' Drive, Minneapolis, MN 55417, USA

## Abstract

*Background*. An active device that downregulates abdominal vagal signalling has resulted in significant weight loss in feasibility studies. *Objective*. To prospectively evaluate the effect of intermittent vagal blocking (VBLOC) on weight loss, glycemic control, and blood pressure (BP) in obese subjects with DM2. *Methods*. Twenty-eight subjects were implanted with a VBLOC device (Maestro Rechargeable System) at 5 centers in an open-label study. Effects on weight loss, HbA_1c_, fasting blood glucose, and BP were evaluated at 1 week to 12 months. *Results*. 26 subjects (17 females/9 males, 51 ± 2 years, BMI 37 ± 1 kg/m^2^, mean ± SEM) completed 12 months followup. One serious adverse event (pain at implant site) was easily resolved. At 1 week and 12 months, mean excess weight loss percentages (% EWL) were 9 ± 1% and 25 ± 4% (*P* < 0.0001), and HbA_1c_ declined by 0.3 ± 0.1% and 1.0 ± 0.2% (*P* = 0.02, baseline 7.8 ± 0.2%). In DM2 subjects with elevated BP (*n* = 15), mean arterial pressure reduced by 7 ± 3 mmHg and 8 ± 3 mmHg (*P* = 0.04, baseline 100 ± 2 mmHg) at 1 week and 12 months. All subjects MAP decreased by 3 ± 2 mmHg (baseline 95 ± 2 mmHg) at 12 months. *Conclusions*. VBLOC was safe in obese DM2 subjects and associated with meaningful weight loss, early and sustained improvements in HbA_1c_, and reductions in BP in hypertensive DM2 subjects. This trial is registered with ClinicalTrials.gov NCT00555958.

## 1. Introduction

It has been estimated that approximately 20 million adults in the USA have type 2 diabetes mellitus [1]. Worldwide, the incidence of type 2 diabetes mellitus is now thought to be over 347 million [2]. In the USA alone, it is estimated that it will cost 147 billion dollars to take care of diabetes and its complications [1]. The majority of these individuals are obese. Obesity and type 2 diabetes mellitus (DM2) are closely linked [3]—as body mass index (BMI) increases, there is a weight-dependent increased risk of developing type 2 diabetes [4].

Body weight reduction of just 5% can improve glycemic control in obese type 2 diabetics [5]. Current medical treatments are limited by many factors including subject's compliance and high costs as well as by the inexorable progression of the disease and its complications. Current bariatric surgical procedures such as gastric bypass, biliopancreatic diversion or sleeve gastrectomy have demonstrated a significant beneficial impact on the glucose control even before significant weight loss occurs [6, 7]. However these procedures are invasive with the potential for serious complications and considerable professional and patient aversion. Given the severity of the problem, there is a societal need for a less invasive, effective treatment.

It has been known for a century that the vagus nerve has multiple physiologic functions related to food intake, energy metabolism, and glycemic control [8, 9]. In addition, researchers such as Kral et al. [10] have demonstrated that interrupting or cutting the vagus nerve (vagotomy) can result in meaningful weight loss, satiety, and satiation. Vagotomy is irreversible and can require a secondary gastric outlet procedure in cases of significant gastric retention postvagotomy. In addition, theoretically speaking, permanence may result in compensatory mechanisms that may blunt physiological responses. Therefore, a lower risk, intermittent, and reversible vagal blocking that allows for nerve function recovery would be an attractive alternative. An electrical and reversible vagal blocking device has recently been developed and evaluated (VBLOC Therapy) [11, 12]. This technology was designed to intermittently interrupt neural traffic in the intra-abdominal vagal trunks and has been shown to decrease gastric contractions and pancreatic exocrine secretion in the animal model [13]. 

Retrospective assessment of prior clinical experience with intermittent blocking of the intra-abdominal vagal trunks (VBLOC) in a small number of subjects with DM2 showed improvement in glycemic control in conjunction with weight loss [14]. Significant improvement in blood pressure in subjects with elevated baseline blood pressure has also been reported with VBLOC [15]. These improvements in glycemic control and blood pressure were observed within weeks following initiation of VBLOC therapy and were sustained during the 6-month followup period. 

The aim of this study was to prospectively assess the effect of VBLOC in an open-label, multicenter clinical trial of obese type 2 diabetics. The primary efficacy objectives were improvements in (a) body weight, (b) fasting plasma glucose and HbA_1c_, (c) systolic and diastolic blood pressure, and (d) mean arterial pressure after 12 months of VBLOC Therapy. 

## 2. Materials and Methods

### 2.1. Study Design

This study was a prospective, open-label, and multi-center study to evaluate the safety and efficacy of high frequency electrical algorithms applied to the intra-abdominal vagal trunks in facilitating weight loss and improving glycemic control and blood pressure in type 2 diabetics. Outcomes were compared with subjects' preimplant baseline measurements.

This study was conducted at Instituto Nacional de la Nutricion (INNSZ), Mexico City, Mexico; Trondheim University Hospital, Trondheim, Norway; University Hospital, Basel, Switzerland; Flinders Medical Centre, Adelaide, Australia; and Institute of Weight Control, Sydney, Australia. 

### 2.2. Study Subjects

Device safety and efficacy were assessed during a 12-month study in obese female and male subjects (BMI 30–40 kg/m^2^ inclusive, age 25–60 years inclusive) with type 2 diabetes. Written informed consent was provided from all subjects. The study was approved by local medical ethics committees. General inclusion criteria included prior failure of durable response to medical weight management that involved diet, behavioral modification, and/or pharmacotherapy. Fertile women required contraception and proof of nonpregnancy within 14 days of implant. Relevant exclusion criteria included type 1 diabetes mellitus, smoking cessation within 6 months, weight loss drug therapy within the last 3 months, significant weight loss in the last 12 months (>10% body weight loss), hiatal hernia, the presence of an implanted electrical medical device, or major abdominal surgery, excluding cholecystectomy and hysterectomy. Inclusion criteria for Type 2 diabetes included ≤12-years duration of diabetes, baseline HbA_1c_ levels ≥7% to ≤10%, and absence of significant type 2 diabetes complications, such as nephropathy, retinopathy, neuropathy, or coronary artery disease. Diabetes-related exclusion criteria included insulin dependence and use of GLP-1 receptor agonists. Short-term insulin use was allowed during the perioperative period if needed.

### 2.3. Study Device and Implantation Method

Subjects received a fully implantable Maestro Rechargeable System consisting of two leads, placed laparoscopically. One lead was placed on the anterior trunk and one on the posterior intra-abdominal vagal trunk. These leads were connected to a subcutaneously implanted, rechargeable neuroregulator as previously described [11, 12]. A mobile charger was used for approximately 30 minutes daily to recharge the subcutaneous battery.

### 2.4. Experimental Therapy and Follow-Up Studies

Systems were activated approximately two weeks after implantation. Biphasic pulses at a frequency of 5000 Hz and amplitude from 3 to 8 mA (mode = 6) were applied to block vagal neural impulses [11, 12, 16] with a duty cycle of 5 minutes blocked then 5 minutes unblocked for up to 15 hours daily. The objective was for patients to receive a minimum of 12 hours to a maximum of 15 hours therapy daily depending on patient's response to therapy and daily lifestyle. The patient's therapy delivery algorithm was programmed using a custom-designed software program by the follow-up team. The goal was for the current amplitude to be set at 6 mA nominally and to deliver this amplitude for approximately 14 hours per day on average over the first year. 

All subjects received 17 individual weight management counseling sessions during which basic weight loss and physical activity information was delivered. The initial session was 45 minutes, sessions 2–4 were 30 minutes, and the remaining sessions were 15 minutes long. Only standard weight management materials were used. No support groups, behavioral therapists, or exercise specialists were employed in this trial. General information regarding weight loss, calorie goals, healthy eating strategies, exercise strategies, and record keeping was discussed.

Weight was measured at baseline, weekly through 4 weeks, biweekly to 12 weeks, and monthly to 12 months. HbA_1c_ and fasting plasma glucose (FPG) were measured (ICON Laboratories, Farmingdale, NY, USA) at baseline, 1, 4, and 12 weeks and 6 and 12 months. Blood pressure was measured in triplicate (as described by Pickering et al., 2005) [17], with subjects seated, at 5-minute intervals between measurements using a properly sized cuff (i.e., standard adult size (16 × 30 cm) for arm circumference of 27 to 34 cm or large adult size (16 × 36 cm) for 35 to 44 cm arm circumference) at baseline, 1, 4, and 12 weeks and 6 and 12 months. Hypertension was defined as systolic blood pressures ≥130 mmHg and/or diastolic blood pressures ≥80 mmHg according to the JNC-7 criteria for type 2 diabetics [18]. Waist circumference was measured at the iliac crest (NHANES III Protocol).

Adverse event (AE) inquiries were completed at each visit. Clinical laboratory assessments and 12-lead electrocardiograms (Mayo Medical Laboratories, Rochester, MN, and Quintiles Limited, Berkshire, England) were completed at baseline, implant, device initiation, 4 and 12 weeks, and 6 and 12 months. Medication changes and dose adjustments were recorded at each visit. Neither the surgeon nor the allied health professional from the clinic was involved in any treatment decisions to reduce or cease any medication. 

### 2.5. Calculation of Percent EWL

Ideal body weight was determined [19] by measuring each subject's height and calculating the body weight at BMI of 25.0 for that subject (i.e., ideal body weight (kg) = 25 × height (m)^2^). 

 Next, excess body weight in kg (total body weight at baseline − ideal body weight) was determined, and percent EWL was calculated (weight loss/excess body weight × 100).

### 2.6. Statistical Analysis

Baseline characteristics, and demographics were summarized using descriptive statistics. Mean values with standard errors of the mean (SEM) summarized continuous variables while frequency distributions were summarized as categorical (including binary) variables. 

Mean excess weight loss (EWL %) and changes in HbA_1c_, FPG, and blood pressure (mean arterial pressure, systolic blood pressure, and diastolic blood pressure) at 1, 4, and 12 weeks, and 6 and 12 months were assessed using two-sided, one sample *t*-tests. Changes in waist circumference at 12 weeks and 12 months were assessed using a two-sided, one sample *t*-test. The rate of occurrence of AEs was analyzed. 

### 2.7. Additional Statistical Modeling of Glycemic Control Parameters

To determine if there was a relationship between the reduction observed in FPG or HbA_1c_ at 12 months compared to baseline values, linear regression techniques were employed. The general linear model (PROC GLM in SAS) was used to analyze change in glucose from baseline to 12 months after implantation for FPG and HbA_1c_ separately. The following three models were run using change in either parameter from baseline to 12 months as the response variable. The significance level for interaction was 0.1 (two-sided). The significance level for both baseline FPG or HbA_1c_ and % EWL at 12 months was 0.05 (two-sided). The three models included the following

 Model (1): baseline FPG or HbA_1c_ and % EWL at 12 months were used as independent variables; the interaction between baseline FPG or HbA_1c_ and % EWL at 12 months was also included in the model to determine whether the relationship between change in FPG or HbA_1c_ and baseline glucose is dependent on % EWL at 12 months. If interaction was not significant, it was excluded from the model.
 Model (2): baseline FPG or HbA_1c_ and % EWL at 12 months were used as independent variables (no interaction term was included).
 Model (3): only % EWL at 12 months was used as an independent variable.

Models 2 and 3 were used to determine whether % EWL alone could explain reduction in FPG and HbA_1c_ from baseline to 12 months after implantation. The R-squared values for models 2 and 3 were compared to determine if the FPG or HbA_1c_ reduction observed at 12 months was primarily due to weight loss or if the FPG or HbA_1c_ level at baseline is also an important factor (i.e., do subjects with higher baseline FPG or HbA_1c_ have a greater change in FPG or HbA_1c_ at 12 months?).

## 3. Results

### 3.1. Participants and Demographics

A total of 28 subjects were enrolled (17 females and 11 males; mean age 51 ± 2 years; mean BMI 37 ± 1 kg/m^2^). Twenty-six subjects completed 12 months of followup, whose demographics were 17 males and 9 females, mean age of 51 ± 2 years, and BMI of 37 ± 1 kg/m^2^. Two of the subjects did not attend the 12-month visit but did not drop out of the study which is currently ongoing. All subjects continue to be followedup to assess safety and efficacy. 

### 3.2. Safety

All procedures were completed laparoscopically, there were no surgical complications, and all patients were discharged either on the same day or on the following day as consistent with normal hospital policy. There were no deaths or operative complications. In addition there were no unanticipated adverse device effects. One serious adverse event (SAE) occurred in this trial. The SAE was neuroregulator site pain as a result of its placement directly on the ribcage, above the costal margin, proximally mid-axillary line. The discomfort was eliminated by moving the neuroregulator inferior to the costal margin on the upper left abdominal wall.

### 3.3. Weight Loss

Percent EWL at various time periods following device activation is shown in Table 1. Mean % EWL at 12 months was 25 + 4% (*P* < 0.0001). Average hours of therapy delivery per day over the 12 months were 14 ± 0.1 hours with 6 ± 0.1 mA average current amplitude demonstrating that all subjects received similar algorithms. BMI reduction at 12 months was 3.0 ± 0.4 kg/m^2^. Weight loss at 12 months was 8.4 ± 1.4 kg (*P* < 0.0001).

### 3.4. Changes in Glycemic Control

HbA_1c_ was reduced at all time periods from a baseline of 7.8 ± 0.2% (mean ± SEM, Table 1). Mean % HbA_1c_ reduction at 12 months was 1.0 ± 0.2% (*P* = 0.02, Table 1). FPG was also reduced at all time periods from a baseline of 151 ± 7 mg/dL (Table 1). Mean FPG reduction at 12 months was 28 ± 8 mg/dL (*P* = 0.02, Table 1).

At baseline, of the 26 subjects with a 12 month visit, seventeen subjects took one diabetes medication, 8 subjects took two or more diabetes medications, and one took none. By the 12-month visit, three subjects discontinued their diabetes medication, and six subjects decreased the dose of medications while thirteen subjects had no change. Four subjects increased diabetes medications. 

### 3.5. Change in Blood Pressure

Statistically significant reductions in SBP, DBP, and MAP from baseline were observed at many time points after implantation in all subjects (Table 1). SBP fell to 121 mmHg by 1 week after activation with a further reduction that was sustained throughout the 12-month period. Likewise, DBP fell below 80 mmHg by 1 week which was sustained over the entire evaluation period. Finally, MAP fell to 91 mmHg by 1 week after activation and the reduction was sustained. Elevated blood pressure (SBP ≥ 130 and/or DBP > 80 mmHg) was documented in 15 of the obese diabetic subjects. A significantly reduced mean arterial blood pressure (MAP) in subjects with elevated systolic and/or diastolic blood pressure to nonhypertensive levels from a baseline of 100 ± 2 mmHg was observed at all time points (*P* = 0.04, Table 1). Significant reductions were also observed in subjects with elevated SBP at one time point (Table 1) from a baseline of 140 ± 4 mmHg (*P* = 0.03). However, at all time points the mean SBP was reduced to below 130 mmHg. Finally, significant reductions were observed in subjects with elevated DBP at all time points from a baseline of 88 ± 2 mmHg (*P* = 0.009, Table 1).

### 3.6. Statistical Modeling of Glycemic Control Parameters

**Figure 1:**
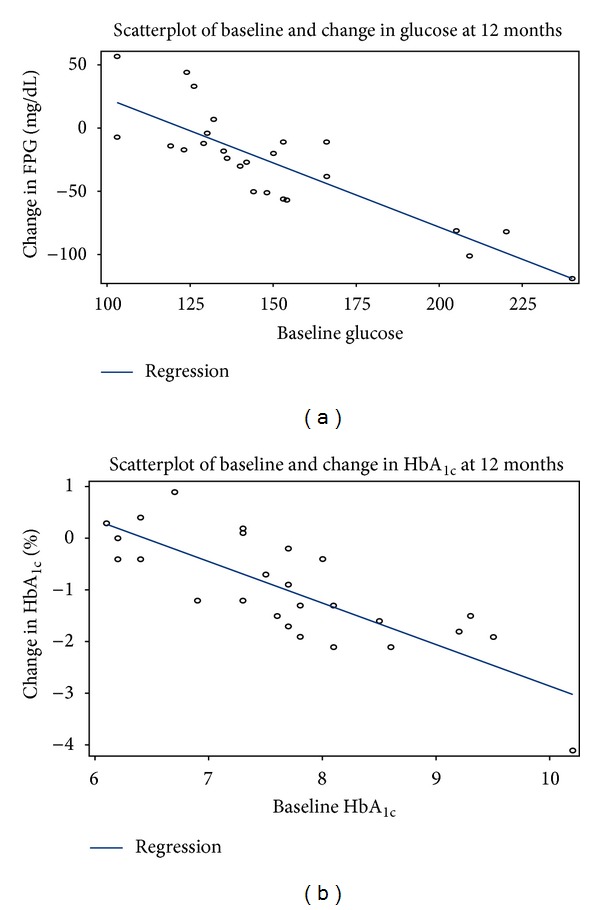
(a) Relationship of change in fasting plasma glucose (mg/dL) at 12 months compared to baseline, *P* < 0.0001, *r* = −0.85. (b) Relationship of change in HbA_1c_ (%) at 12 months compared to baseline *P* < 0.0001, *r* = −0.8.

Linear regression results indicated that FPG (mg/dL) reductions (Figure 1(a)) and HbA_1c_ reductions (Figure 1(b)) were positively associated with baseline preoperative levels (*P* < 0.0001).

Model 1 showed that the positive relationship between reduction in FPG at 12 months and its baseline value was not dependent on % EWL achieved (interaction *P* = 0.18). Model 1 also showed that the positive relationship between reduction in HbA_1c_ at 12 months and its baseline value was not dependent on % EWL achieved (interaction *P* = 0.34).

Model 2 showed that overall improvements of FPG could not be explained by % EWL alone. R-squared values for the models with % EWL only were 0.4. When baseline values for FPG were added to the models with % EWL values, the R-squared increased to 0.8 indicating that both baseline values of FPG and % EWL were needed to explain the reduction in this parameter. An additive effect of weight loss and VBLOC Therapy on reduction in FPG was observed.

In a similar manner, Model 3 showed that overall improvements of HbA_1c_ could not be explained by % EWL alone. R-squared for the models with % EWL only were 0.5. When baseline values for HbA_1c_ were added to the model with % EWL, the R-squared increased to 0.85 indicating that both baseline values of HbA_1c_ and % EWL were needed to explain the reduction in this parameter. An additive effect of weight loss and VBLOC Therapy on reduction in HbA_1c_ was observed.

### 3.7. Additional Findings of Clinical Interest

Waist circumference decreased by 8 ± 1 cm, 9 ± 2 cm, and 11 ± 2 cm at 12 weeks and 6 and 12 months, respectively (*P* < 0.001, baseline = 120 ± 2 cm, *n* = 23).

## 4. Discussion

This open-label prospective trial of VBLOC therapy in obese type 2 diabetic patients demonstrated that VBLOC therapy was safe and effective for achieving clinically significant weight loss and improving both DM2 and high blood pressure. Additionally, there were no significant adverse events and the therapy was well tolerated by all of the patients. 

The ramifications of the increase in the incidence and prevalence of obesity and DM2 in the USA and throughout the world are becoming well understood as they affect both budgets and the public health of nations. Currently, over two-thirds of Americans are overweight and over one-third are obese [20]. In addition, approximately 8% of US adults and 19% of adults over 65 years of age are diabetic [1]. The more sobering fact is that the coexistence of type 2 diabetes and obesity increases the risk of developing hypertension and cardiovascular disease [21] thereby increasing morbidity and mortality [22]. There is also a good reason to believe that the prevalence of these conditions will continue to increase around the globe [20].

While current bariatric surgical procedures have been shown to be highly successful for improving (and even forcing into remission) these devastating chronic illnesses, [23, 24] too few candidates undergo these operative procedures. In the USA, it is believed that less than 1% to 2% of prospective candidates undergo bariatric surgery. This disconnection between an efficacious treatment and potential candidates is multifactorial. It includes factors such as medical insurance restrictions, prejudices against the obese, the fear of the perioperative risks, and long-term consequences of these procedures. In short, it is clear that for many obese patients, conventional bariatric surgery is not a viable option. This phenomenon has created a significant need for new and novel interventions that are safer, effective for both weight control and DM2, and offer fewer long-term complications. A safe, more simple, and efficacious therapeutic option would have the potential to increase the number of potential patients able to undergo bariatric surgery.

One such new technology is vagal nerve activity blocking with a patterned electrical impulse delivered to the intra-abdominal nerve trunks (VBLOC therapy). Based on the growing understanding of the vagus nerve in energy regulation, appetite, and glucose regulation, VBLOC therapy is increasingly showing itself to be promising [11, 12]. In this trial, VBLOC therapy was studied in a cohort of obese patients (mean BMI 37 ± 1 kg/m^2^) with DM2. Clinically significant weight loss of 25% EWL occurred by 12 months. Early improvements in glycemic control were observed. HbA_1c_ levels were reduced to 7.1% from a baseline of 7.8% by 4 weeks and fell to 6.7% by 12 weeks. This reduction was maintained at 12 months. Twenty-two of 26 subjects (85%) were found to be able to maintain, decrease, or discontinue their diabetes medications during the first 12 months while achieving improved glucose control. The Look AHEAD study showed that 33% of patients of the control group who were given standard diabetes support and education and who were on no medications at baseline started taking diabetes medications over the first 12 months [25]. 

 The final analysis performed was to analyze the level of HbA_1c_ reduction achieved with at least 5% total body weight loss (Table 2) to compare to the published weight loss literature in obese subjects with type 2 diabetes. A published weight loss study in subjects with type 2 diabetes showed that subjects who achieved at least 5% total body weight loss (BWL) experienced HbA_1c_ reductions of 0.53 percentage points [26]. In contrast, the data from the VBLOC-DM2 study indicate that subjects who achieved at least 5% BWL experienced significantly greater HbA_1c_ reductions of 1.4 percentage points at 12 months.

Improvements in blood pressure were also observed in the subjects with elevated blood pressure with no adverse changes in normotensive subjects. The addition of VBLOC therapy to an existing medication regimen resulted in significant improvements in glucose regulation in the DM2 cohort and blood pressure control in the hypertensive patients while allowing over 80% of subjects to reduce or maintain their diabetes medication. All medication decisions were made by the patient's primary physician and not by the investigators. Lastly, subjects significantly decreased their waist circumferences by over 11 cm at 12 months. Since waist circumference is a surrogate marker for visceral adiposity, it appears that weight loss by VBLOC is producing the “right” type of weight loss [3].

 The results are encouraging and create a solid foundation for a larger study. It is acknowledged by the investigators, that a follow-up period of only 12 months does not guarantee long-term efficacy. There have been many investigations of novel weight loss interventions that demonstrated promising results for 6 to 12 months, only to lose effectiveness over time [27]. However the results of this trial at 12 months do not suggest any loss of efficacy (Table 1). Currently there are VBLOC therapy studies with 5-year followup underway.

While a mean excess weight loss of approximately 25% is low relative to some other conventional bariatric surgical procedures (e.g., gastric bypass, sleeve gastrectomy, and biliopancreatic diversion), VBLOC therapy has substantially fewer complications. The safety of the device and VBLOC therapy in this trial was excellent, as it was observed in previous trials using VBLOC therapy [11, 12]. The perioperative complications are dramatically less severe and less frequent than those seen with other bariatric operations.

An important observation with VBLOC Therapy was that the improvements of DM2 and hypertension were noted shortly after activation of the device. This early benefit and the stability of the improvement with the continued weight loss over time would suggest that the mechanisms of action may be, at least in part, independent of the weight loss. This is supported by the data modeling presented. These data suggest that vagal nerve blocking has beneficial physiologic effects on appetite and energy regulation. Similar observations have also been noted after Roux-en-Y gastric bypass, where improvements in DM2 often occur within days of surgery. Several studies on the potential mechanisms involved in the rapid control of type 2 diabetes in obese patients after certain surgical interventions have shown that exclusion of the duodenum and the upper part of the jejunum and the more rapid emptying of ingested nutrients into the distal ileum induce significant changes in gastrointestinal hormones such as incretins that are involved in insulin secretion and glucose regulation [28–31]. However, the mechanism of action may also be vagally mediated. Bernal-Mizrachi et al. [32] demonstrated in rodents that the interruption of hepatic afferent vagal pathways prevented glucocorticoid-induced insulin resistance, suggesting a strong vagal role in glycemic control. Further studies will hopefully better elucidate this finding. Similarly, as the vagus is a major nerve of the parasympathetic nervous system, it would not be surprising to observe that it may also be involved in central parasympathetic-sympathetic afferent-efferent control loops that can account for the reduction in blood pressure in hypertensive patients. If validated, modulating the vagus nerve for the treatment of metabolic diseases can open a new direction for surgical research and possibly patient care. 

 There were a number of limitations to this study. The study did not include a control group. The design of the trial was intentional since there is ample literature concerning this population in both surgical and medical treatment studies; it was thought that a control group was unnecessary for this pilot study. Studies evaluating devices, operative procedures, diets, and medications for weight loss have been criticized and it has been suggested that the placebo effect may have been responsible for some or all of the weight loss outcome. For several reasons, we believe that this was not the case with this investigation. Firstly, the weight loss was maintained over the entire 12 months. Secondly, the weight loss was greater than that typically seen in placebo subjects. In the SHAPE Trial evaluating an implantable gastric electrical stimulator, the placebo effect was 11% EWL [33]. This placebo group of subjects was carefully screened for inclusion by a bariatric psychologist and during the study participated in a rigorous dietary program that included monthly group meetings. Additionally, a recent meta-analysis of the effect of dietary counseling for weight loss reported that, on average, dietary counseling resulted in a net loss of approximately 2 BMI units over the first 12 months of the intervention compared to usual care [34]. In addition, studies which included subjects with diabetes showed that diabetics had about 50% less weight loss than nondiabetic subjects [34]. Importantly, VBLOC Therapy studied in these obese type 2 diabetic patients resulted in a 3 kg/m^2^ reduction in BMI at 12 months. Lastly the study included a small number of subjects. Further followup of these patients will hopefully reveal continued efficacy.

## 5. Conclusion

VBLOC was safe, effective and well tolerated in obese DM2 subjects and associated with clinically meaningful weight loss, as well as early and sustained improvements in glucose control and reductions in BP in hypertensive DM2 subjects.

## Figures and Tables

**Table 1:** % EWL and change in glycemic parameters (mg/dL) and blood pressure (mmHg) from baseline over 12 months.

Parameter	Time following device activation—change from baseline						*P* value
	Baseline	1 week	4 weeks	12 weeks	6 months	12 months	
EWL %, *n* = 28	0	9 ± 1	14 ± 2	21 ± 3	24 ± 4	25 ± 4	<.0001
HbA_1c_ (%), *n* = 28	7.8 ± 0.2	−0.3 ± 0.1	−0.7 ± 0.1	−0.9 ± 0.2	−0.9 ± 0.2	−1.0 ± 0.2	.02
FPG (mg/dL), *n* = 28	151 ± 7	−21 ± 6	−19 ± 7	−27 ± 8	−29 ± 8	−28 ± 8	.01
MAP, all subjects (mmHg), *n* = 28	95 ± 2	−4 ± 2	−5 ± 2	−5 ± 2	−8 ± 2	−3 ± 2	.04^#^
SBP, all subjects (mmHg), *n* = 28	125 ± 2	−3 ± 3	−7 ± 3	−6 ± 3	−8 ± 3	−4 ± 3	.04^∧^
DBP, all subjects (mmHg), *n* = 28	80 ± 2	−5 ± 2	−4 ± 2	−4 ± 2	−8 ± 2	−2 ± 2	.04^&^
MAP elevated (mmHg), *n* = 15	100 ± 2	−7 ± 3	−9 ± 3	−9 ± 2	−13 ± 2	−8 ± 3	.04
SBP elevated (mmHg), *n* = 8	140 ± 4	−10 ± 9	−12 ± 10	−13 ± 5	−16 ± 8	−12 ± 9	.03*
DBP elevated (mmHg), *n* = 12	88 ± 2	−10 ± 2	−10 ± 3	−9 ± 1	−14 ± 2	−10 ± 3	.009

^#^At 1, 4, and 12 weeks and 6 months; ^∧^at 4 weeks, 12 weeks and 6 months; ^&^at 1 week, 12 weeks and 6 months; *at 12 weeks.

FPG: fasting plasma glucose, MAP: mean arterial pressure, SBP: systolic blood pressure, and DBP: diastolic blood pressure.

**Table 2:** Change in HbA_1c_ (%) at various time points after VBLOC therapy initation in trial subjects with at least 5% BWL*.

Visit	*n*	ΔHbA_1c_ (%) mean ± SEM	Minimum	Maximum
1 Wk	4	−0.8 ± 0.2	−1.2	−0.3
4 Wk	11	−1.0 ± 0.2	−2.5	0.0
12 Wk	18	−1.2 ± 0.2	−3.2	0.3
6 Mo	16	−1.1 ± 0.3	−3.1	0.5
12 Mo	16	−1.4 ± 0.2	−4.1	0.0

*BWL: total body weight loss.

## References

[1] Wild S, Roglic G, Green A, Sicree R, King H (2004). Global prevalence of diabetes: estimates for the year 2000 and projections for 2030. *Diabetes Care*.

[2] Danaei G, Finucane MM, Lu Y (2011). National, regional, and global trends in fasting plasma glucose and diabetes prevalence since 1980: systematic analysis of health examination surveys and epidemiological studies with 370 country-years and 2*·*7 million participants. *The Lancet*.

[3] Maggio CA, Pi-Sunyer FX (1997). The prevention and treatment of obesity: application to type 2 diabetes. *Diabetes Care*.

[4] Must A, Spadano J, Coakley EH, Field AE, Colditz G, Dietz WH (1999). The disease burden associated with overweight and obesity. *Journal of the American Medical Association*.

[5] Fujioka K (2010). Benefits of moderate weight loss in patients with type 2 diabetes. *Diabetes, Obesity and Metabolism*.

[6] Mingrone G, Panunzi S, De Gaetano A, Guidone C, Iaconelli A, Leccesi L (2012). Bariatric surgery versus conventional medical therapy for type 2 diabetes. *The New England Journal of Medicine*.

[7] Schauer PR, Kashyap SR, Wolski K, Brethauer SA, Kirwan JP, Pothier CE (2012). Bariatric surgery versus intensive medical therapy in obese patients with diabetes. *The New England Journal of Medicine*.

[8] Guyton AC, Hall JE (2000). *Textbook of Medical Physiology*.

[9] Pardo JV, Sheikh SA, Schwindt GC (2008). Chronic vagus nerve stimulation for treatment-resistant depression decreases resting ventromedial prefrontal glucose metabolism. *NeuroImage*.

[10] Kral JG, Paez W, Wolfe BM (2009). Vagal nerve function in obesity: therapeutic implications. *World Journal of Surgery*.

[11] Camilleri M, Toouli J, Herrera MF, Kow L, Pantoja JP, Billington CJ (2009). Selection of electrical algorithms to treat obesity with intermittent vagal block using an implantable medical device. *Surgery for Obesity and Related Diseases*.

[12] Camilleri M, Toouli J, Herrera MF (2008). Intra-abdominal vagal blocking (VBLOC therapy): clinical results with a new implantable medical device. *Surgery*.

[13] Tweden KS, Sarr MG, Bierk MD, Camilleri M, Kendrick ML, Knudson MB (2006). Vagal blocking for obesity control (VBLOC): studies of pancreatic and gastric function and safety in a porcine model. *Surgery For Obesity and Related Diseases*.

[14] Herrera MF, Brancatisano R, Keller U, Kulseng B, Toouli J, Brancatisano A (2009). Intermittent vagal blockade with an implantable device improves glycemic control in obese subjects with type 2 diabetes. *Surgery For Obesity and Related Diseases*.

[15] Herrera MF, Kow L, Pantoja JP, Knudson MB, Tweden KS, Wilson RR (2009). VBLOC and improvements in co-morbidities in obese subjects during weight loss. *Obesity Surgery*.

[16] Tweden KS, Anvari M, Bierk MD, Billington CJ, Camilleri M, Honda CN (2006). Vagal blocking for obesity control (VBLOC): concordance of effects of very high frequency vagal blocking currents at the neural and organ levels using two pre-clinical models. *Gastroenterology*.

[17] Pickering TG, Hall JE, Appel LJ (2005). Recommendations for blood pressure measurement in humans and experimental animals: part 1: blood pressure measurement in humans. *Circulation*.

[18] (2003). The seventh report of the Joint National Committee on the prevention, detection, evaluation, and treatment of high blood pressure (JNC-7). *Publication no.*.

[19] Bray GA, Bouchard C, Church TS (2009). Is it time to change the way we report and discuss weight loss?. *Obesity*.

[20] Fryar CD, Carroll MD, Ogden CL Prevalence of overweight and obesity among adults: United States, trends 1960–1962 through 2009-2010 (Health E-Stat). http://www.cdc.gov/nchs/data/hestat/obese/obese99.htm.

[21] Hubert HB, Feinleib M, McNamara PM, Castelli WP (1983). Obesity as an independent risk factor for cardiovascular disease: a 26-year follow-up of participants in the Framingham Heart Study. *Circulation*.

[22] Lew EA, Garfinkel L (1979). Variations in mortality by weight among 750,000 men and women. *Journal of Chronic Diseases*.

[23] Dixon JB, O’Brien PE, Playfair J (2008). Adjustable gastric banding and conventional therapy for type 2 diabetes: a randomized controlled trial. *Journal of the American Medical Association*.

[24] Schauer PR, Burguera B, Ikramuddin S (2003). Effect of laparoscopic Roux-En Y gastric bypass on type 2 diabetes mellitus. *Annals of Surgery*.

[25] Wing RR, Bahnson JL, Bray GA (2010). Long-term effects of a lifestyle intervention on weight and cardiovascular risk factors in individuals with type 2 diabetes mellitus: four-year results of the look AHEAD trial. *Archives of Internal Medicine*.

[26] Fujioka K, Seaton TB, Rowe E (2000). Weight loss with sibutramine improves glycaemic control and other metabolic parameters in obese patients with type 2 diabetes mellitus. *Diabetes, Obesity and Metabolism*.

[27] Elder KA, Wolfe BM (2007). Bariatric surgery: a review of procedures and outcomes. *Gastroenterology*.

[28] Scopinaro N, Papadia F, Camerini G, Marinari G, Civalleri D, Franco AG (2008). A comparison of a personal series of biliopancreatic diversion and literature data on gastric bypass help to explain the mechanisms of resolution of type 2 diabetes by the two operations. *Obesity Surgery*.

[29] Korner J, Bessler M, Inabnet W, Taveras C, Holst JJ (2007). Exaggerated glucagon-like peptide-1 and blunted glucose-dependent insulinotropic peptide secretion are associated with Roux-en-Y gastric bypass but not adjustable gastric banding. *Surgery for Obesity and Related Diseases*.

[30] Patriti A, Facchiano E, Sanna A, Gullà N, Donini A (2004). The enteroinsular axis and the recovery from type 2 diabetes after bariatric surgery. *Obesity Surgery*.

[31] Bose M, Oliván B, Teixeira J, Pi-Sunyer FX, Laferrère B (2009). Do incretins play a role in the remission of type 2 diabetes after gastric bypass surgery: what are the evidence?. *Obesity Surgery*.

[32] Bernal-Mizrachi C, Xiaozhong L, Yin L (2007). An afferent vagal nerve pathway links hepatic PPAR*α* activation to glucocorticoid-induced insulin resistance and hypertension. *Cell Metabolism*.

[33] Shikora SA, Bergenstal R, Bessler M (2009). Implantable gastric stimulation for the treatment of clinically severe obesity: results of the SHAPE trial. *Surgery for Obesity and Related Diseases*.

[34] Dansinger ML, Tatsioni A, Wong JB, Chung M, Balk EM (2007). Meta-analysis: the effect of dietary counseling for weight loss. *Annals of Internal Medicine*.

